# Claims-Based Frailty Among Newly Admitted Long-stay Nursing Home Residents

**DOI:** 10.1093/geroni/igaf122.4326

**Published:** 2025-12-31

**Authors:** Kailin Xu, Chan Mi Park, Brianne Olivieri-Mui, Sarah Berry, Dae Hyun Kim, Sandra Shi

**Affiliations:** Hebrew SeniorLife, Boston, Massachusetts, United States; Hebrew SeniorLife & Harvard Medical school, Boston, Massachusetts, United States; Northeastern University, Boston, Massachusetts, United States; Hebrew SeniorLife & Harvard Medical school, Boston, Massachusetts, United States; Hebrew SeniorLife, Boston, Massachusetts, United States; Hebrew SeniorLife, Boston, Massachusetts, United States

## Abstract

Admission to a long-stay nursing home (LNH, defined as ≥ 30 accumulative days within an episode) is a sentinel event for older adults, often signaling limited life expectancy or increased risk of adverse health outcomes. However, the prevalence of frailty and its prognostic value at LNH admission remain poorly characterized. In a 7% random sample of fee-for-service Medicare beneficiaries from 2015 to 2019, we identified 66,407 new LNH residents. Frailty was measured using the claims-based frailty index (CFI) on claims in the 12 months prior to LNH admission and categorized as non-frail (CFI < 0.25), mild frailty (0.25-0.34), moderate frailty (0.35-0.44) or severe frailty (≥0.45). We examined the association of frailty category with mortality, hospitalization, ED visits, injurious falls, and pressure ulcers over one-year follow-up, using Cox proportional hazards or Fine–Gray competing-risk models adjusted for age, sex, and dual eligibility. Frailty was present in 77.1% at LNH admission, with 46.7% mild frailty, 25.6% moderate frailty, and 4.8% severe frailty. One-year risk of mortality increased with frailty: 20.4% in non-frailty, 26.3% in mild frailty, 30.6% in moderate frailty, and 33.5% in severe frailty. Differences in mortality persisted when stratified by age and sex. Compared with non-frail beneficiaries, those with severe frailty had higher rates of mortality (HR [95% CI], 2.0 [1.8, 2.1]), hospitalization (2.8 [2.6–2.9]), ED visits (1.8 [1.6–1.9]), injurious falls (1.2 [0.9–1.5), and pressure ulcers (3.9 [3.4–4.4]). These results highlight that a claims-based frailty assessment can be useful for prognostication and risk stratification among newly admitted LNH residents.

